# Experimental Study of Comprehensive Performance Analysis Regarding the Dynamical/Mechanical Aspects of 3D-Printed UAV Propellers and Sound Footprint

**DOI:** 10.3390/polym17111466

**Published:** 2025-05-25

**Authors:** Florin Popișter

**Affiliations:** Department of Design Engineering and Robotics, Faculty of Industrial Engineering, Robotics and Production Management, Technical University of Cluj-Napoca, B-dul Muncii 103-105, 400641 Cluj-Napoca, Romania; florin.popister@muri.utcluj.ro

**Keywords:** 3D printing, propeller, fuse filament fabrication, digital light processing, sound, temperature

## Abstract

The present study evaluates the viability of fabricating unmanned aerial vehicle (UAV) propellers using fused filament fabrication (FFF), with an emphasis on low-cost, desktop-scale production. The study’s backdrop is the recent adoption of UAVs and advancements in additive manufacturing. While the scope targets accessibility for individual and small-scale users, the results have broader implications for scalable UAV propulsion systems. The research was conducted within an experimental UAV development framework aimed at optimizing propeller performance through strategic material selection, geometrical design optimization, and additive manufacturing processes. Six propeller variants were manufactured using widely available thermoplastic polymers, including polyethylene terephthalate glycol-modified (PETG) and thermoplastic polyurethane (TPU), as well as photopolymer-based propellers fabricated using vat photopolymerization, also known as digital light processing (DLP). Mechanical and aerodynamic characterizations were performed to assess the structural integrity, flexibility, and performance of each material under dynamic conditions. Two blade configurations, a toroidal propeller with anticipated aerodynamic advantages and a conventional tri-blade propeller (Gemfan 51466-3)—were comparatively analyzed. The primary contribution of this work is the systematic evaluation of performance metrics such as thrust generation, acoustic signature, mechanical strength, and thermal stress imposed on the electrical motor, thereby establishing a benchmark for polymer-based propeller fabrication via additive manufacturing. The findings underscore the potential of polymeric materials and layer-based manufacturing techniques in advancing the design and production of UAV propulsion components.

## 1. Introduction

Unmanned aerial vehicles (UAVs), commonly known as drones, have been used in certain fields for some time. However, their widespread adoption has accelerated in recent years due to the increasing availability of commercial models and the growing affordability of drone construction for enthusiasts. This has led to the continuous development of new systems and technologies, driven both by manufacturers seeking to expand their market share through product innovation and by skilled users experimenting with new configurations to enhance efficiency. This growth is evident across various sectors, including aerial photography, agriculture, environmental monitoring, and logistics [1].

Among the key components of a drone, the propeller plays a crucial role in determining aerodynamic efficiency, maneuverability, and overall stability [2]. Traditional drone propellers, typically made from plastic or reinforced composite materials, provide the performance required for commercial and industrial applications. However, they can be expensive and may cause sourcing delays, particularly for hobbyists and small-scale users.

The limitations of conventionally manufactured propellers pose challenges for domestic users, particularly those involved in DIY drone construction or small-scale manufacturing. While traditional propeller materials are durable, they often have manufacturing constraints and accessibility issues that make them less practical for home users. These challenges could potentially be addressed through alternative materials and production methods [3]. The emergence of 3D printing technology, particularly fused filament fabrication (FFF), offers an opportunity to produce custom propellers at a lower cost than traditional manufacturing methods while also allowing for greater design flexibility and faster prototyping cycles [4].

This study aims to evaluate the feasibility of using FFF and vat photopolymerization techniques to manufacture drone propellers from polymer-based materials, specifically PETG, TPU, and resin [5]. By focusing on materials and methods accessible to hobbyists, the research seeks to explore whether 3D-printed propellers can serve as a cost-effective and readily available alternative to conventionally manufactured counterparts [6]. The study is particularly relevant to small-scale users and enthusiasts who wish to experiment with propeller designs and materials without requiring extensive resources.

The evaluation will assess the suitability of PETG, TPU, and H200 hard-tough resin for 3D-printed drone propellers by analyzing key performance characteristics, including thrust, structural strength, noise levels, and thermal performance. Additionally, two propeller designs, the toroidal and 3-bladed models, will be compared to determine the impact of material properties and geometry on efficiency, stability, and noise levels [7,8,9,10].

The research contributes to the growing body of knowledge on accessible manufacturing for drone components, particularly for domestic and enthusiast applications, by investigating the potential of 3D-printed polymer propellers. The findings are expected to provide valuable recommendations for hobbyists and small-scale users interested in manufacturing their own propellers. If FFF and vat photopolymerization prove to be viable methods, they could enhance the accessibility of drone technology, enabling users to explore customized designs and material properties suited to specific operational needs [11].

Propellers are crucial to drone performance, affecting noise, flight time, and thrust [12,13]. Vertical take-off and landing (VTOL) maneuvers require high-velocity airflow directed vertically to produce the reactive force needed for lift. Take-off, being the most demanding maneuver, requires substantial thrust from the motors.

To understand airflow around propeller blades, the Coandă effect is relevant. It occurs when airflow adheres to a surface, linked to Bernoulli’s principle, which describes the inverse relationship between fluid velocity and pressure. As flow speed increases, pressure decreases in adjacent areas [14]. This principle, used in aviation, explains how wing shapes generate lift by creating pressure differences, similar to helicopter blades and drone propellers.

The design of propeller blades also impacts drone noise. Drone propeller noise is primarily caused by three factors: rotational noise, impulsive noise, and broadband noise. Rotational noise arises from blade interactions with air, influenced by blade geometry and loading conditions [15]. Impulsive noise is generated by abrupt airflow changes, such as blade tip vortices, causing pressure fluctuations. Broadband noise is associated with turbulent airflow around the blades [15].

To evaluate the feasibility of 3D-printed propellers and provide data for informed decisions, six propeller probes were produced. These included two pairs, with materials selected for affordability and accessibility: TPU, PETG, and resin. The probes were divided into two groups: one with toroidal geometry and the other with a common 3-bladed design. This resulted in six distinct probes.

In the context of drone propellers, this comparison suggests that FFF may be the preferred method to produce larger, load-bearing components. This is due to the accessibility, cost, and capacity for functional prototypes associated with this technique. While vat photopolymerization may be less practical for high-stress components, it could be valuable in the creation of precise, low-weight parts where surface finish and dimensional accuracy are of paramount importance. This is particularly the case if hybrid designs incorporating both methods can be explored.

The present study is concerned with three specific materials. Each of the materials, namely PETG, TPU and H200 hard-tough resin, possesses distinctive properties that are conducive to the 3D printing of drone propellers.

## 2. Materials and Methods

Additive manufacturing (AM), or 3D printing, is a transformative technology that enables the production of complex geometries often unattainable with traditional manufacturing methods [16]. AM allows for design flexibility, waste reduction, and rapid prototyping, which benefit industries from biomedical to aerospace [17]. Recently, 3D printing has become more accessible to hobbyists, thanks to lower equipment and material costs, particularly in processes like FFF. This accessibility has led to experimental applications in areas such as drone component fabrication, where users can design and test propellers with varied geometries and materials.

**Fused filament fabrication (FFF)** is a popular form of AM, where thermoplastic filament is heated and extruded layer by layer to form structures. It is affordable, easy to use, and offers material flexibility, making it accessible to both industrial and personal users [18]. The mechanical properties and precision of FFF parts depend on process parameters like layer height, nozzle temperature, print speed, and infill density. For example, higher infill density improves strength but increases material use and print time [19]. While suitable for functional components under moderate stress, FFF has limitations in dimensional precision and surface finish compared to other AM methods [20]. FFF offers a cost-effective, scalable approach for drone propeller prototyping, benefiting both domestic users and small-scale production.

**Polyethylene terephthalate glycol (PETG)** is a copolyester with good strength, chemical resistance, and greater ductility than PET, making it ideal for functional parts under moderate stress [21]. Compared to PLA, PETG is less brittle and has better thermal stability, making it a practical choice for parts exposed to moderate temperatures. Its balance of strength, durability, and printability makes it well suited for drone propellers needing resilience and flexibility under load [22].

**Thermoplastic polyurethane (TPU)** is a flexible polymer known for its elasticity, durability, and impact resistance, making it suitable for parts requiring flexibility [23]. However, TPU’s softness presents printing challenges [24]. Its properties are beneficial for absorbing shocks and vibrations, making it useful for propellers exposed to impacts. However, its elasticity may limit its use in high-speed applications, where deformation could occur.

**Vat photopolymerization (resin printing)** uses UV light to cure liquid photopolymer resin layer by layer, producing objects with fine detail and smooth surfaces [25]. This process, including SLA and DLP, provides high accuracy and isotropic mechanical properties but results in brittleness and lower impact resistance than thermoplastics. It is better suited for applications requiring precision and fine detailing, such as dental and jewelry fabrication [26]. In drone applications, resin printing’s high precision and smooth surface can improve propeller performance, especially in smaller components [27].

A comparison of **FFF and vat photopolymerization** reveals trade-offs between cost, accuracy, mechanical properties, and ease of use. FFF offers robust, cost-effective prints with materials like PETG and TPU, which are durable for mechanical applications [28]. However, the layer-by-layer extrusion creates anisotropic properties, making parts weaker along the *z*-axis [29]. By contrast, vat photopolymerization provides near-isotropic properties, offering consistent mechanical performance across axes but at a higher material cost and with greater brittleness [30]. Additionally, resin printing requires post-processing, adding complexity and time, which can hinder large-scale or rapid prototyping compared to FFF. Table 1 compares PETG, TPU, and H200 hard-tough resin in terms of mechanical strength, flexibility, print quality, and cost-effectiveness [31,32,33,34,35,36,37,38].

During the printing process, parameters such as layer height, print speed, and temperature settings (see Table 2) were adjusted to optimize print quality and adherence to design specifications.

**Figure 1:**
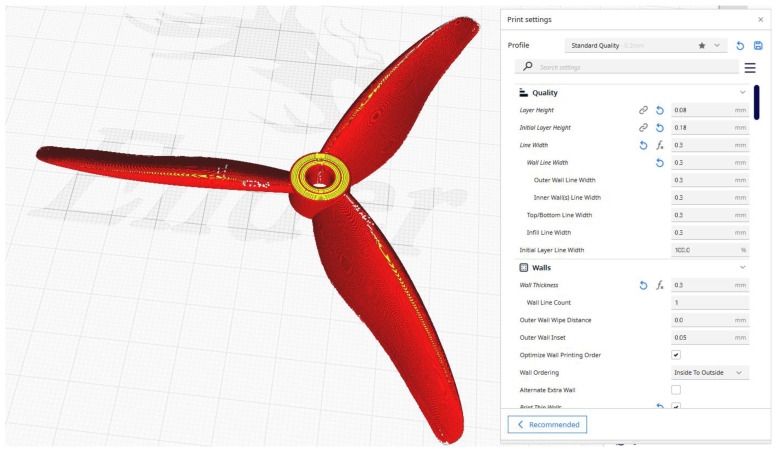
Slicing process of a propeller probe.

The post-processing solution employed for the FFF pieces was “UltiMaker Cura 5.7”, which offers a comprehensive range of print parameters that can be tailored to achieve optimal print quality, particularly in the context of intricate geometries, as observed in the selected propellers. Figure 1 illustrates the slicing parameters of one of the probes.

**Figure 2:**
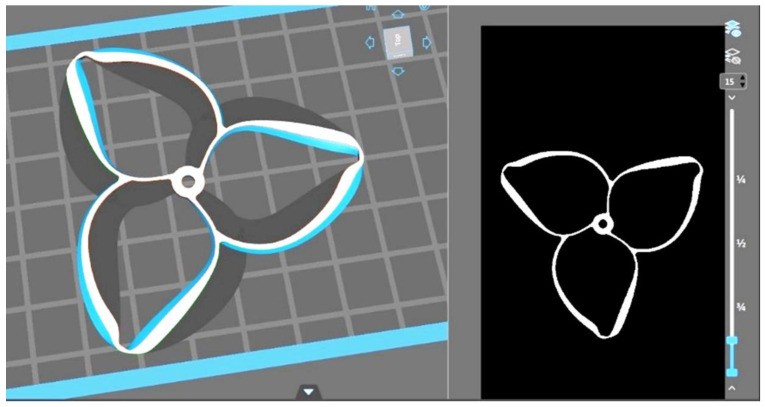
The post-processing of the resin toroidal propeller.

To print the DLP-specific propellers, the post-processing software used was “Chitubox”. This allowed the modification of specific parameters to achieve the produced part in correspondence with the CAD model. Figure 2 depicts a screen capture of the post-processing procedure of one of the 3D-printed propellers.

The PETG filament was the easiest to work with, after making minor adjustments within the post-processor, while the printing process of the resin propellers was more difficult due to the necessary curing stage.

From a dimensional perspective, both propeller models were designed based on 3D models featuring an outer diameter of 120 mm and a socket diameter of 5 mm. These specifications ensured proper mounting on the shaft of the BLDC motor, minimizing the risk of vibrations and facilitating secure attachment.

**Figure 3:**
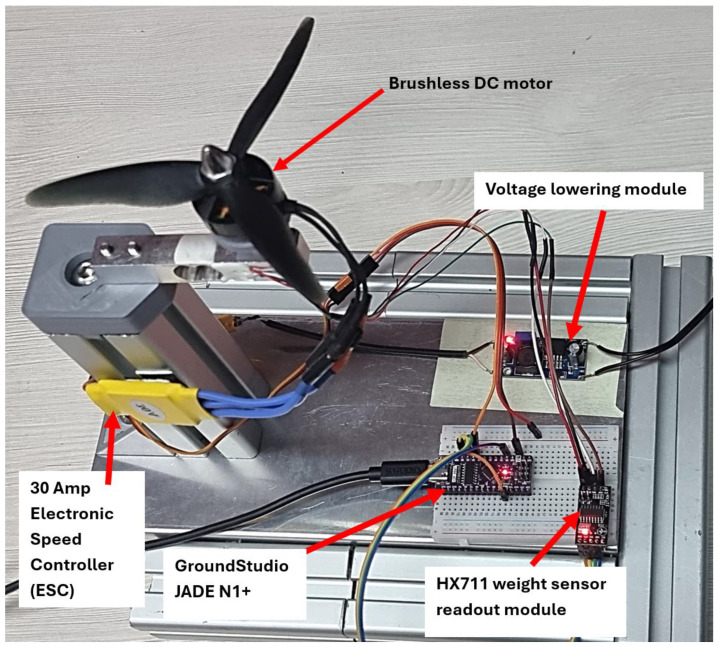
Experimental stand meant for measuring the thrust force generated by the propellers.

One of the main concerns in using propellers produced by non-conventional means as an alternative to those offered on the market is closely linked to the main function of such parts, which is to generate thrust efficiently to achieve satisfactory mobility characteristics for UAVs. In this context, the study presents an experimental stand, Figure 3, for the precise measurement of thrust forces that the proposed probes can achieve.

To accurately quantify the thrust force, the stand must perform several key functions. Propeller emulation was achieved using a brushless DC motor (MT2204) designed for drone applications. Operating at a maximum RPM/V value of 2300 and input voltage of 7.4 V, the motor reached a maximum rotational speed of 17,020 RPM.

The motor was controlled by a 30 Amp electronic speed controller (ESC) powered by a 7.4 V regulated supply. The control signal was sent from a “GroundStudio JADE N1+” microcontroller, programmed via “Arduino IDE” for serial communication, which transmitted commands and displayed results.

The thrust force was measured using a DIXSZE load-cell sensor, capable of measuring up to 5 kg, connected to a control module. Calibration was performed at the beginning of each session by recording the sensor’s zero reading and applying a pre-measured weight using a pulley system. The weights, measured with high precision, totaled 512.09 g, including a deformable wire. The sensor’s calibration library allowed for input of any accurately measured weight.

## 3. Results and Discussion

The experimental procedure for each of the six propeller probes is as follows:

1. The probe is mounted onto the motor shaft.
2. Calibration is performed by recording the zero value.
3. The pre-measured weight is applied, and the measurement is recorded.
4. The motor operates at 40% of its maximum RPM, and the thrust is recorded.
5. The RPM is increased to 60%, and the corresponding thrust is recorded.
6. The motor is deactivated, and the process is repeated for the next propeller.

The motor operated at 40% and 60% of its RPM range to compare the thrust generated by the six propellers. The focus was on comparing performance differences, not achieving maximum thrust. Operating at these speeds ensured safety and avoided exceeding the recommended motor RPM. The results are summarized in Table 3. The standard deviation within these measurements was 0.02 N. It is to be mentioned that the presented values are the average upon a sampling size of 5 measurements.

The results confirmed the hypothesized superior thrust performance of the toroidal propellers, with an average thrust value 0.26 Newtons higher than that of the classic geometry propellers in the Gemfan geometry. This was due to the different geometry of the toroidal model compared to the 3-bladed structure.

Among the materials tested, the resin propellers outperformed both the TPU and PETG models. The enhanced surface finish of resin likely contributed to improved aerodynamic efficiency, offering better overall performance. To assess the propeller resistance to axial loads from the thrust force and identify any potential risks to users, all six probes underwent compression tests. The key finding included that the TPU propellers showed a linear relationship between load and deformation in probes 1 and 4.

The resin classic 3-bladed propeller (probe 5) exhibited bending at the junction between the blades and the circular part. However, compared to the TPU model, the resin model was more rigid, reaching a tensile extension load of 16.18 N. Probe 3 demonstrated an intriguing behavior. Initially, it resembled probe 5, which reached a maximum load of 69.25 N. However, probe 3 showed higher load resistance, peaking at 49.49 N. At this point, the resin polymer’s elastic modulus caused cracks in the propeller structure, ultimately compromising its integrity. The best performance was seen in probe 2, the PETG toroidal propeller. The load increased rapidly with extension, peaking at 140 N, marking the onset of plastic deformation. The load eventually reached 178.82 N at a tensile extension of 27.61 mm, although significant structural damage occurred beyond this point. The highest extension under maximum tensile load occurred in the TPU propellers, particularly probes 1 and 4, with the classic 3-bladed model extending 19.018 mm, while the toroidal model reached 12.206 mm.

**Figure 4:**
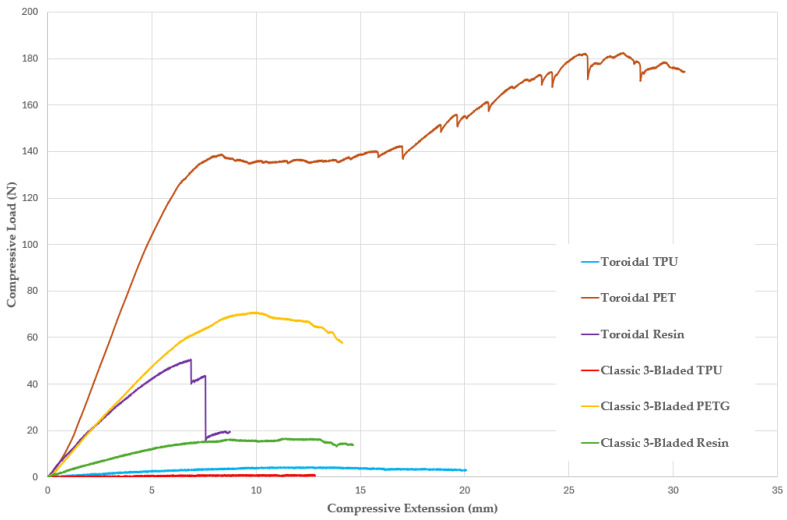
Graphical representation of the load/extension function corresponding to the 6 probes.

The results of the trials are presented in the form of a graph, depicted in Figure 4, where each specimen corresponds to one of the probes as follows:

1. *Specimen 1*—Toroidal—TPU
2. *Specimen 2*—Toroidal—PETG
3. *Specimen 3*—Toroidal—Resin
4. *Specimen 4*—classic 3-bladed propeller—TPU
5. *Specimen 5*—classic 3-bladed propeller—PETG
6. *Specimen 6*—classic 3-bladed propeller—Resin

Table 4 illustrates the difference in performance, with the TPU toroidal propeller (probe 1) withstanding a maximum tensile load of 4.10 Newtons, compared to only 0.88 Newtons for the classic 3-bladed model (Probe 4). This difference was attributed to the geometry of the blade.

**Figure 5:**
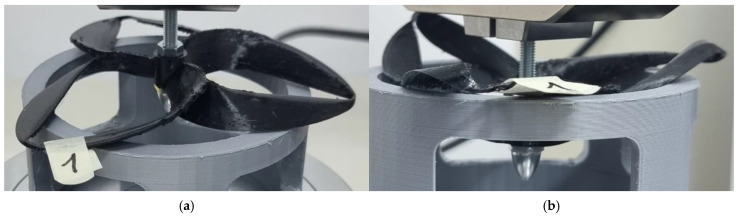
Probe 1 initial state (**a**) and probe 1 deformation (**b**)—TPU was the material used for the toroidal geometry.

In Figure 5, depicting the trial of the first specimen, the mode of deformation is particularly noteworthy. Due to the flexibility of the TPU filament, the blade extremities at the conjunction points tended to bend toward one another, as observed in the nearest pair of blades, or tilt upward, as seen in the pair farthest from the camera. The elastic deformation of the blades allowed the propeller to revert to its original shape after the trial, rendering it reusable.

**Figure 6:**
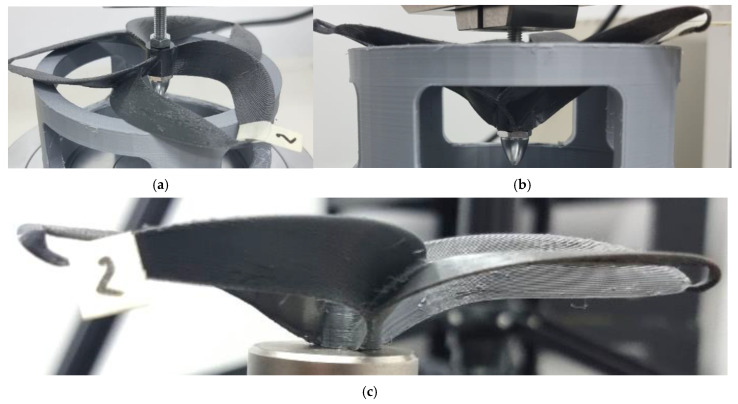
Probe 2 initial state (**a**), probe 2 deformation detail (**b**), and probe 2 after the test (**c**)—PETG was the material used for the toroidal geometry.

The PETG toroidal propeller, represented by probe 2, demonstrated the highest load resistance during the test. However, as observed in Figure 6b,c, the propeller did not revert to its original shape. It exhibited deformation along the *z*-axis, as well as at the conjoining tips of the blades, where the stresses acted similarly to those noted in the case of probe 1.

**Figure 7:**
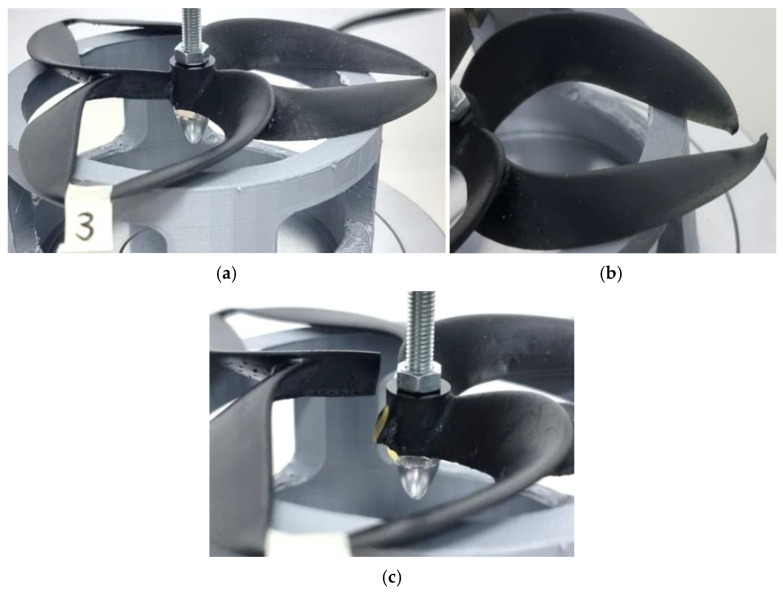
Probe 3 initial state (**a**), probe 3 initial failure point (**b**), and probe 3 second failure point (**c**)—resin was the material used for the toroidal geometry.

The resin specimen of the toroidal model, identified as specimen 3, sustained significant damage during the test, resulting in the formation of two distinct fracture points. Figure 7b highlights the section of the toroidal geometry that consistently demonstrated the highest fragility across all three toroidal propellers tested. The first failure occurred at the narrow section connecting one of the blade pairs, leading to a structural split. The second fracture emerged near the mounting hub, as depicted in Figure 7c. This failure may be attributed to the aggressive pitch angle of the propeller blades, which likely contributed to an unfavorable stress distribution in this region.

**Figure 8:**
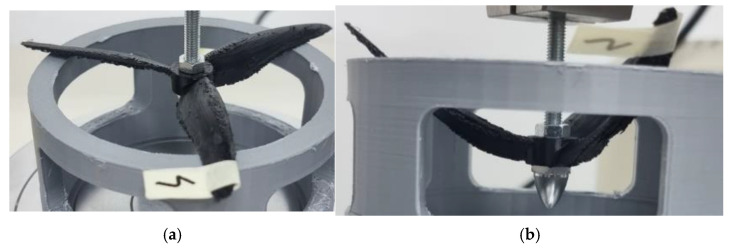
Probe 4 initial state (**a**) and probe 4 deformation (**b**)—TPU was the material used for the classic 3-bladed propeller geometry.

**Figure 9:**
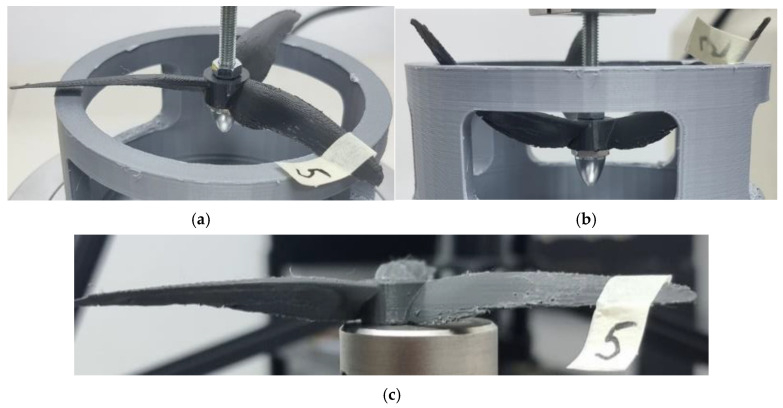
Probe 5 initial state (**a**), probe 5 deformation (**b**), and probe 5 after test (**c**)—PETG was the material used for the classic 3-bladed propeller geometry.

**Figure 10:**
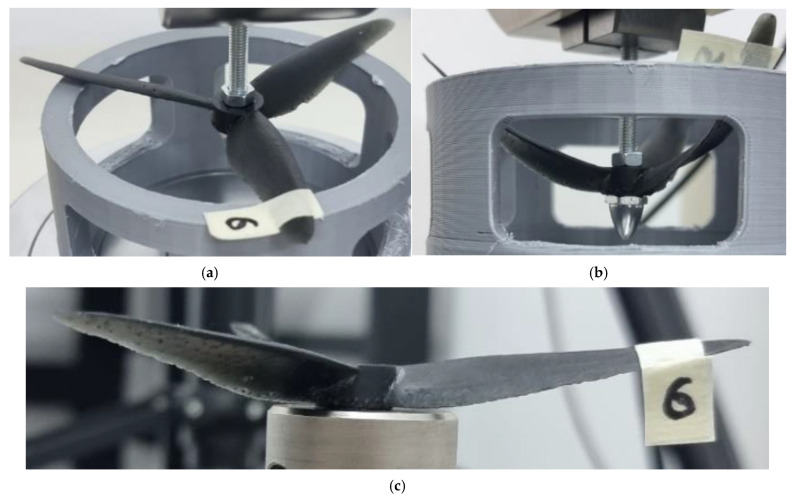
Probe 6 initial state (**a**), probe 6 deformation (**b**), and probe 6 after test (**c**)—resin was the material used for the classic 3-bladed propeller geometry.

The analysis of Figure 8, Figure 9 and Figure 10 revealed consistent behavior among the three specimens of the classic 3-bladed propeller geometry. All three specimens underwent deformation without exhibiting notable levels of resistance. The most significant observation was that none of the specimens experienced catastrophic deformation within the plastic domain, nor did they present any fracture points. The only observable permanent deformation is evident in Figure 10c, which shows a side view of the resin specimen displaying an upward angular offset of the blades.

The experiment’s analysis revealed key findings that support existing theories while offering new insights. Among the configurations tested, the PETG toroidal propeller showed the highest resistance to axial load, closely mimicking the thrust force generated. However, it demonstrated potential for deformation under higher thrust values. Importantly, no fractures occurred in either geometry, even at the most vulnerable point of the toroidal model, at the junction of the blade pairs. This suggests enhanced safety, reducing the risk of projectile formation or structural failure during propeller contact.

**Figure 11:**
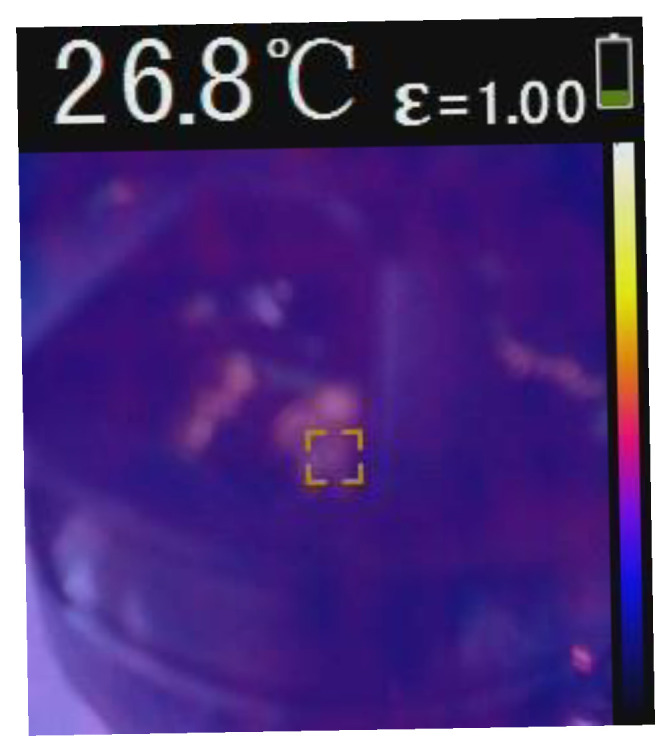
Reference value of winding temperature at the start of each trial.

The experiment also involved measuring the motor winding temperature after each operational cycle with the six different propellers. To ensure accuracy, ambient temperature and RPM were controlled. A thermal camera and digital laser infrared sensor were used for temperature measurements. The sensor’s characteristics included an ambient temperature range of −20 to 85 °C and a field of view of 33 °C. The initial motor temperature, measured before starting after a period of inactivity, served as the baseline for each trial, as shown in Figure 11.

The experiment consisted of trials for each of the six propeller configurations, with each trial following the steps outlined below:

1. The temperature of the motor windings is measured using the thermal camera. If the winding temperature matches the reference value, the trial can proceed.
2. A timer is set for 5 min, and the first propeller probe is mounted and securely fastened onto the motor’s shaft.
3. Simultaneously, the timer and the motor are started, with the motor running at 60% of its maximum rotational speed.
4. The motor is stopped when the timer ends, and the temperature of the windings is immediately measured using the thermal camera.
5. The propeller is removed, and the motor’s temperature is gradually brought back to the reference value.
6. The experiment is repeated with a different propeller.

Table 5 presents the recorded heat values measured after each experiment, with each probe model displaying its corresponding heatmap and temperature readings.

A comparative analysis of the propeller models at the material level indicated that the toroidal configuration consistently resulted in lower and more uniform motor winding temperatures across all tested materials, with a maximum of 28.8 °C, while PETG showed a minor deviation. By contrast, the classic 3-bladed design exhibited greater thermal variability, with resin imposing the least thermal load (29.3 °C) and TPU the highest (30.1 °C). Overall, resin emerged as the most efficient material and the toroidal design as the least thermally demanding configuration. When considering the small differences between obtained values, such as the cases of the TPU material and resin material in terms of toroidal geometry, fluctuation of the ambient temperature should be considered while analyzing the obtained results.

The acoustic signature of propellers, especially toroidal ones, is an area of significant research, particularly in contexts such as urban environments, surveillance, or stealth applications. For domestic use, the acoustic characteristics of 3D-printed propellers should be carefully evaluated to mitigate noise pollution and potential auditory health effects [39].

**Figure 12:**
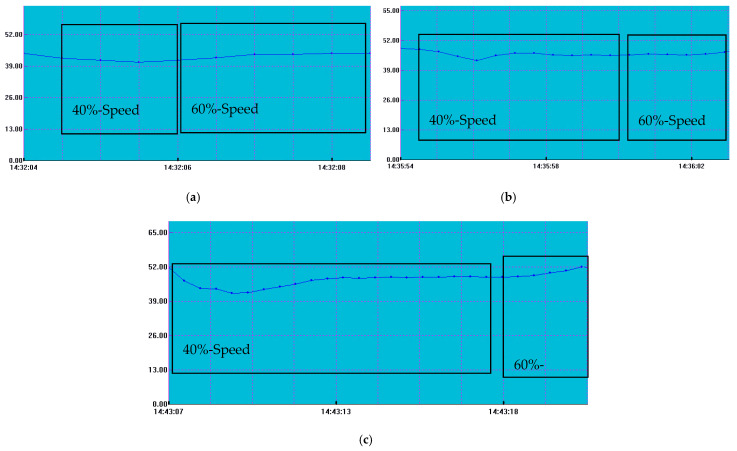
The measured sound profiles of the toroidal ***propeller probes***: (**a**) TPU, (**b**) PETG, and (**c**) resin.

**Figure 13:**
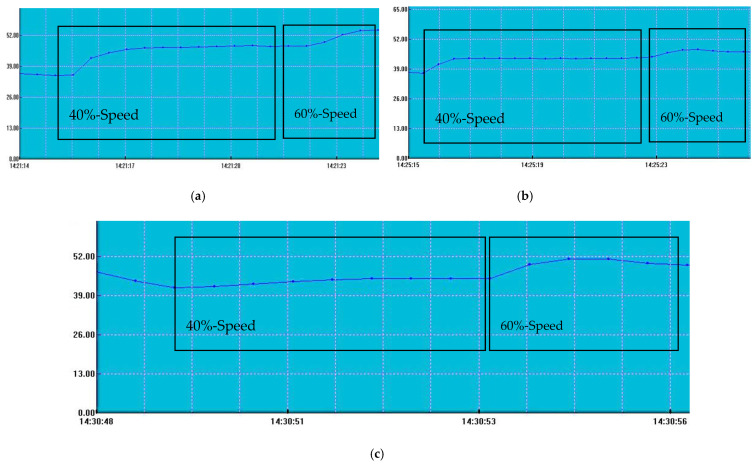
The measured sound profile of the *3-bladed propeller probes*: (**a**) TPU, (**b**) PETG, and (**c**) resin.

A professional sonometer (PCE-322A model) was used at a distance of 125 cm to measure the sound signature of each propeller at both 40% (approximately 6800 RPM) and 60% (approximately 10,200 RPM) of the maximum RPM. Measurements were sustained long enough to capture stable sound levels. The data, presented in Figure 12 and Figure 13, are shown graphically, with the vertical axis representing the sound amplitude in decibels and the horizontal axis depicting the time of measurement.

The analysis of the results revealed no significant change in sound amplitude as the rotational speed increased for the TPU toroidal propeller, similar to the heat test results for the same model. This can likely be attributed to the material’s deformation factor.

The classic 3-bladed propellers exhibited greater sensitivity to rotational speed changes, particularly in terms of sound output. At 40% RPM, they produced lower sound levels compared to the toroidal models, but at higher RPM values, the noise levels were comparable. The greatest increase in noise was noted in the TPU classic 3-bladed propeller, which also had the highest overall decibel rating. The resin toroidal propeller followed closely behind. Despite general expectations that toroidal propellers produce lower sound levels, they performed worse than the classic 3-bladed propellers, likely due to the larger contact area of the blades. While toroidal propellers are generally known for their quieter operation, they tend to have a larger wing surface compared to traditional propeller designs.

The acoustic superiority of the toroidal design stemmed primarily from the merged blade tips, which prevented vortex formation in this area, thus reducing noise emission compared to classic propellers. This distinction highlighted the different sources of sound in the two designs, each operating independently.

During the experiments, several factors were controlled to ensure accurate and reliable results. These included maintaining an environmental temperature of 20 ± 1 °C in accordance with ISO 1:2022 [40], minimizing background noise to ensure that sound measurements exceeded 45 dB above ambience noise, as per ISO 3382-1:2009 [41], and acclimatizing the propeller probes for at least 48 h prior to testing.

## 4. Conclusions

This present study investigated two propeller geometries for UAV applications: the conventional 3-bladed propeller, selected for its established aerodynamic performance and widespread adoption, and the toroidal propeller, chosen for its purported advantages in noise attenuation, enhanced safety, and potential thrust gains. The classic 3-bladed design was utilized as a control model representative of typical multirotor configurations, thereby extending the generalizability of the experimental results. By contrast, the toroidal geometry served as an innovative alternative, offering an opportunity to explore unconventional aerodynamic behaviour.

Three widely accessible and cost-effective polymer materials—thermoplastic polyurethane (TPU), polyethylene terephthalate glycol (PETG), and photopolymer resin—were selected based on their compatibility with consumer-grade additive manufacturing processes. Each material brings distinct benefits: TPU’s flexibility contributes to safety and durability, PETG provides a balanced mechanical profile, and resin yields superior surface finishes that can influence aerodynamic efficiency.

Six propeller prototypes were fabricated and tested, isolating the influence of material composition and blade geometry on key performance indicators. These included thrust output, acoustic emissions, and thermal/mechanical stress imparted on brushless motors. The study revealed that surface roughness significantly impacts noise levels, with smoother finishes associated with lower sound signatures. Notably, the TPU toroidal propeller demonstrated consistent acoustic behaviour across rotational speeds, while the classic 3-bladed variants exhibited increasing noise with higher RPMs. The resin toroidal propeller produced the highest decibel levels, highlighting a trade-off between material properties and acoustic performance.

In addition, the study introduced an evaluation framework for quantifying the mechanical and thermal load on motors during operation. The results indicated that the 3-bladed propellers imposed greater dynamic stress on the motor than the toroidal configurations. Among the materials tested, the resin-based propellers showed lower motor loads, particularly in the toroidal design, although this did not translate into clear advantages in noise suppression.

The principal contribution of this research lies in its detailed comparative analysis of polymer-based propellers fabricated via additive manufacturing, elucidating the complex interplay between material selection, blade geometry, and propulsion system performance. These findings offer actionable insights for optimizing UAV propeller design, with implications for achieving quieter operation, reduced motor wear, and enhanced overall efficiency in 3D-printed propulsion systems.

## Figures and Tables

**Table 1:** Comparison of mechanical and practical properties of PETG, TPU, and H200 hard-tough resin filaments for 3D printing applications.

Attribute	PETG	TPU	H200 Hard-Tough Resin
Tensile Strength [MPA]	67.07	21–36	45.16–49.17
Impact Strength [kJ/m^2^]	70	120	17–35
Flexural Strength [MPa]	80	60–97	133.39
Modulus of Elasticity [GPa]	1.71	2.127	0.841–0.917
Cost	20–30 €/kg	40–70 €/kg	70–100 €/L

**Table 2:** Printing parameters of the propeller in accordance with the materials and manufacturing techniques.

Parameter	Creality Ender 3 S1 Pro-TPU	Creality Ender 3 S1 Pro-PETG	AnyCubic Photon-S
Layer Height [mm]	0.18	0.18	0.05
Wall Line Count	4	4	
Line Width [mm]	0.5	0.5	
Top/Bottom Thickness [mm]	0.18	0.18	
Infill [%]	100	100	100
Lifting Speed [mm/min]			180
Lift Distance [mm]			6
Retract Speed [mm/min]			180
Exposure Time [s]			6
Resolution [px]			X: 1440, Y:2560
Printing temperature [ C]	230	250	
Print Speed [mm/s]	50	50	
Fan Speed [%]	100	50	

**Table 3:** The mean values of thrust generated by each propeller at 40% and 60% of the achievable RPM.

Probe		Measured Thrust Values	
		*Case of 40% rpm*	*Case of 60% rpm*
Toroidal propeller	TPU	2.9 N	3.1 N
	PETG	2.7 N	3.4 N
	Resin	2.6 N	4.4 N
Classic 3-bladed propeller	TPU	2.9 N	3.0 N
	PETG	2.6 N	3.0 N
	Resin	3.0 N	4.1 N

**Table 4:** Numerical results of the tensile extension trials.

	Specimen Label	Maximum Tensile Load (N)	Stress at Max. Compressive Load (MPa)	Tensile Ext. at Max. Load (mm)
1	1	4.10	0.20228	12.20601
2	2	178.82	8.83064	27.61233
3	3	49.49	2.44414	6.87214
4	4	0.88	0.04352	19.01836
5	5	69.25	3.41991	14.95876
6	6	16.18	0.79889	17.06898

**Table 5:** Measured temperature of each propeller probe after a temporally predefined cycle of use.

Propeller Model	Values of the Measured Temperatures [°C]		
	TPU	PETG	RESIN
**Toroidal propeller**	28.9 °C	29 °C	28.8 °C
**Classic 3-bladed propeller**	30.1 °C	29.8 °C	29.3 °C

## Data Availability

Data are contained within the article.
